# Acoustic–Seismic Mixed Feature Extraction Based on Wavelet Transform for Vehicle Classification in Wireless Sensor Networks

**DOI:** 10.3390/s18061862

**Published:** 2018-06-07

**Authors:** Heng Zhang, Zhongming Pan, Wenna Zhang

**Affiliations:** College of Artificial Intelligence, National University of Defense Technology, Changsha, Hunan 410073, China; chungmingpan@nudt.edu.cn (Z.P.); zwna@nudt.edu.cn (W.Z.)

**Keywords:** feature extraction, wavelet coefficient energy ratio (WCER), hierarchical clustering, vehicle classification, wireless sensor networks (WSNs)

## Abstract

An acoustic–seismic mixed feature extraction method based on the wavelet coefficient energy ratio (WCER) of the target signal is proposed in this study for classifying vehicle targets in wireless sensor networks. The signal was decomposed into a set of wavelet coefficients using the à trous algorithm, which is a concise method used to implement the wavelet transform of a discrete signal sequence. After the wavelet coefficients of the target acoustic and seismic signals were obtained, the energy ratio of each layer coefficient was calculated as the feature vector of the target signals. Subsequently, the acoustic and seismic features were merged into an acoustic–seismic mixed feature to improve the target classification accuracy after the acoustic and seismic WCER features of the target signal were simplified using the hierarchical clustering method. We selected the support vector machine method for classification and utilized the data acquired from a real-world experiment to validate the proposed method. The calculated results show that the WCER feature extraction method can effectively extract the target features from target signals. Feature simplification can reduce the time consumption of feature extraction and classification, with no effect on the target classification accuracy. The use of acoustic–seismic mixed features effectively improved target classification accuracy by approximately 12% compared with either acoustic signal or seismic signal alone.

## 1. Introduction

Wireless sensor networks (WSNs) consist of nodes capable of sensing, signal processing, and communicating. In addition to object detection, also of interest are the inherent properties of control and activation in WSNs [1]. One application of WSNs is the classification of moving vehicles in the interested area, which is a precondition for further decision-making in military operations. Moreover, vehicle classification is an important signal processing task and has found widespread civilian applications, such as intelligent transportation systems [2] and real-time traffic surveillance [3].

Signal feature extraction is the basis of vehicle classification and significantly affects the results of classification. Feature extraction methods of target signals applied to WSNs can be classified into two categories: Data fusion algorithms and algorithms based on a special sensor signal.

The feature extraction algorithm, based on data fusion, requires at least two signal sequences of the vehicle. The Haar discrete wavelet transform (DWT)-based method [4] is a vehicle feature extraction method based on data fusion. This method was proposed after combining the multi-sensor data fusion method with the wavelet transform (WT) method. This method employs a maximum entropy measure to determine significant wavelet coefficients. Features are obtained by calculating the energy of coefficients grouped around the competing clusters. Li et al. [5] proposed a multiple sensor information fusion method for vehicle type recognition based on the wireless geomagnetic sensor network. A maximum likelihood estimation method is used to perform vehicle feature waveform information fusion to improve vehicle type recognition accuracy. The feature in, feature out [6] process is another novel fusion method for achieving a joint feature vector estimate based on multiple sensors. The output of this process is fused feature vectors based on the single-source feature vector of an observed object provided by each sensor in the distributed embedded system.

The proposed algorithms based on the data fusion method [7,8] utilize target signals acquired from multi-sensors in WSNs to enhance the accuracy of target classification, but they require greater communication between nodes in a group or in a cluster and consume more energy. However, as hardware and software resources of nodes in WSNs are limited, the complexity and amount of resources required by the algorithms should be considered first. Despite the satisfactory performance demonstrated by a classification-algorithm-based fusion method [9], its high resource requirement has prompted researchers to consider feature extraction methods based on a special sensor signal.

Feature extraction algorithms based on a special sensor signal often use target features in the frequency domain or wavelet domain of their acoustic, seismic, or magnetic signals. The improved time-encoded signal processing algorithm [10] is a feature extraction method based on the acoustic signal of a special sensor. This method was proposed to ameliorate the time-encoded signal processing algorithm, which is effective for feature extraction of speech signals, but not suitable for vehicle acoustic signals. This algorithm designs an extensional symbol table with 40 characters according to the characteristic features of the vehicle acoustic signal and, thereafter, uses a support vector machine (SVM) [11] as the classifier to recognize different vehicle types. A fast Fourier transform (FFT)-based feature extraction method [12] was introduced to extract vehicle features in a real-life vehicle tracking sensor network. The extracted features, which were based on the frequency spectrum of the acoustic signals of the target, were used to test three classification algorithms. There have also been studies attempting to use the time-domain features of the target signal to classify vehicles. For example, a time domain harmonics amplitude method [13], which extracts features from the energy of the target signal by estimating the strongest harmonic frequency and the harmonics’ amplitude from a template of the acoustic signal, was developed for vehicle classification in a sensor network.

The algorithms mentioned above utilize the acoustic signal, seismic signal, or magnetic signal of the target acquired by a single sensor node in WSNs to classify the target type. In this paper, we propose a different approach to extract the signal feature based on the acoustic and seismic signals acquired from a single sensor node for vehicle classification. Instead of using a traditional spectral-domain or time-domain method, we use a WT-based method to extract features from acoustic and seismic signals. First, the signal sequence was decomposed into a set of wavelet coefficients by using the à trous algorithm. After the wavelet coefficients of the target acoustic and seismic signals were obtained, the energy ratio of each layer coefficient was calculated and utilized as the feature vector of the target signals. Subsequently, the acoustic and seismic features were merged into an acoustic–seismic mixed feature to improve target classification accuracy.

The rest of the paper is organized as follows. The wavelet coefficient energy ratio (WCER) method, based on wavelet decomposition using the à trous algorithm, is introduced in Section 2. Section 3 introduces the feature vector, simplified using the hierarchical cluster method, and Section 4 introduces the acoustic–seismic mixed feature of the target signal. After briefly reviewing the data source and the DARPA SensIT project [12], and utilizing the subset data provided by this experiment to validate the proposed method, the performance of the method is discussed in Section 5. Section 6 concludes the paper.

## 2. WCER Feature Extraction Method

Wavelet transform is a signal processing technique that represents a transient or non-stationary signal in terms of time and scale distribution, and is an excellent tool for data compression [14], analysis, and denoising [15]. After wavelet composition of the signal, the wavelet coefficients represent the signal components in different frequency bands, making it feasible to differentiate signals of different targets.

### 2.1. Wavelet Decomposition Based on à Trous Algorithm

The wavelet decomposition method and reconstruction method are based on WT. WT decomposes a signal into wavelet coefficients by using a set of base functions [16]. A set of functions gained following basic wavelet function *Ψ*(*t*) were flexed in scale and displaced in the time domain.

(1) $$WT_{x}(a,b)=<x(t),\Psi_{a,b}(t)>=\frac{1}{\sqrt{\left|a\right|}}\int_{+\infty }^{-\infty }x\left(t\right)\Psi^{*}\left(\frac{t-b}{a}\right)dt,\;a,b\in R,a\neq 0$$

where *Ψ*(*t*) is the wavelet “prototype”, which can be considered a bandpass function [17], *a* is the contraction–expansion or scale factor, and *b* is the displacement factor.

Instead of continuously varying the parameters, as in the case of the continuous wavelet transform [18], which contains a large amount of redundant information, we can analyze the signal with a smaller number of scales with a varying number of translations at each scale. This is the DWT process [19]. The à trous algorithm [20] is one of the most concise methods to implement the fast DWT. The algorithm utilizes the equivalent translocation property of the z-transform and performs a convolution operation of orthogonal filter coefficients and signal to calculate the wavelet coefficients of the signal sequence after inserting a certain number of zeros between the coefficients of the orthogonal filter.

**Figure 1:**
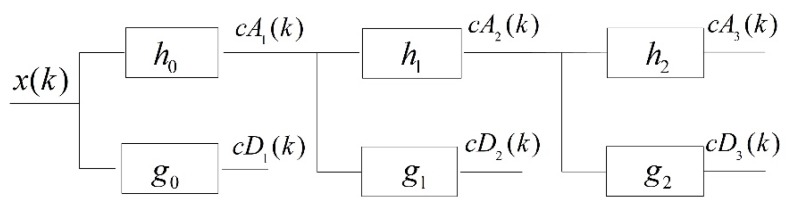
Three-level wavelet decomposition algorithm based on the à trous algorithm, *h_j_* represents *h*_0_ up-sampled by 2*^j^* and *g_j_* represents *g*_0_ up-sampled by 2*^j^*.

Figure 1 shows the scheme of the three-level wavelet decomposition based on the à trous algorithm. *x*(*k*) is the signal sequence and *h_j_* and *g_j_* are the orthogonal filter bank coefficients of the (*j −* 1) level in the wavelet decomposition process of the signal. *cA*_j_(*k*) indicates the wavelet approximation coefficients of the *j*th level and represents the low-frequency components of the current wavelet decomposition of the signal. *cD*_j_(*k*) indicates the wavelet detail coefficients of the *j*th level and represents the high-frequency components of the current wavelet decomposition of the signal. *h_j_* represents *h*_0_ up-sampled by 2*^j^*, while *g_j_* represents *g*_0_ up-sampled by 2*^j^*. When using the à trous algorithm to decompose the target signal, the orthogonal filter bank coefficients of the upper level are interpolated to obtain the filter coefficients of the current level first and, thereafter, the input sequence of the current level is convolved with the orthogonal filter banks to calculate the wavelet coefficients. The approximation coefficient obtained at the previous level is utilized as the input signal of the next level. The formulas for the calculation of *cA*_j_(*k*) and *cD*_j_(*k*) are as follows:

(2) $$cA_{j}\left(k\right)=cA_{j-1}\left(k\right)⊗h_{j}=\overset{k}{\underset{l=1}{\sum }}h_{j}\left(l\right)cA_{j-1}\left(k-l+1\right),\;h_{j}\left(l\right)=0\;whilel>L_{j}$$

(3) $$cD_{j}\left(k\right)=cD_{j-1}\left(k\right)⊗g_{j}=\overset{k}{\underset{l=1}{\sum }}g_{j}\left(l\right)cD_{j-1}\left(k-l+1\right),\;g_{j}\left(l\right)=0\;whilel>L_{j}$$

where ⊗ represents a convolution operation; *k* = 1, 2, …, *K* and *K* is the length of the signal sequence; and *j* = 1, 2, …, *J* and *J* is the desired wavelet composition depth. The lengths of the orthogonal filter bank coefficients, *g_i_* and *h_i_*, at each decomposition level are different. *L_j_* represents the length of the orthogonal filter bank coefficients at the *j*th level. *cA*_0_(*k*) is equal to the initial signal sequence, *x*(*k*), when calculating *cA*_1_(*k*) and *cD*_1_(*k*) using Equations (2) and (3), respectively. The length of the result sequence after the convolution operation is *K* + *L_j_* − 1, which indicates that the sequence length after convolution at different levels of wavelet decomposition is not the same. To simplify the calculation, we intercept a sequence of length *K* from the middle of the convolution result sequence at each wavelet decomposition level.

Considering the wavelet decomposition of the first layer as an example, the convolution result of the signal, *x*(*k*), and decomposition high-pass filters, *g*_0_, is the wavelet detail coefficient, *cD*_1_(*k*), and the wavelet approximation coefficient, *cA*_1_(*k*), is obtained using the convolved signal, *x*(*k*), and the decomposition low-pass filters, *h*_0_. *cA*_1_(*k*), is, thereafter, utilized as the input signal of the second level. After this wavelet decomposition algorithm is completed, the signal, *x*(*k*), is decomposed into a set of approximation coefficients and detail coefficients.

### 2.2. Wavelet Coefficients Energy Ratio (WCER) Method

As the wavelet coefficients of the signal correspond to different frequency bands of the target signal with different signal spectrum distribution, the wavelet coefficients of the target signals have different characteristics and can be utilized to extract spectrum features for classification purposes.

The difference on spectrums of different target signals make the energy of the signal component in different frequency bands discrepant, hence, the wavelet coefficients of acoustic and seismic signals of different targets vary. WCER, which can indicate the difference between energies of coefficients, can intuitively reflect the energy difference of signals in different frequency bands. WCER can be expressed as the wavelet detail coefficient energy ratio (WDcer) and wavelet approximation coefficient energy ratio (WAcer), which are defined as follows:

(4) $$WDcer(j)=\frac{PD(j)}{\sum_{j=1}^{J}PD(j)},WAcer(j)=\frac{PA(j)}{\sum_{j=1}^{J}PA(j)}$$

where *J* is the desired wavelet transform depth, *PD*(*j*) represents the *j*th wavelet detail coefficient energy, and *PA*(*j*) represents the *j*th wavelet approximation coefficient energy. The formulas for calculating *PD*(*j*) and *PA*(*j*) are as follows:

(5) $$PD(j)=\overset{K}{\underset{k=1}{\sum }}{\left(cD_{j}(k)\right)}^{2},PA(j)=\overset{K}{\underset{k=1}{\sum }}{\left(cA_{j}(k)\right)}^{2}$$

where *N* is the length of the wavelet coefficient, which is also the length of the signal sequence. After WDcer and WAcer are calculated, the feature of the target signal based on WCER can be obtained. The feature vector of the target signal is denoted as **f**.

(6) f=[WDcer(1),⋯,WDcer(J);WAcer(1),⋯,WAcer(J)]

### 2.3. Signal Feature Extraction

**Figure 2:**
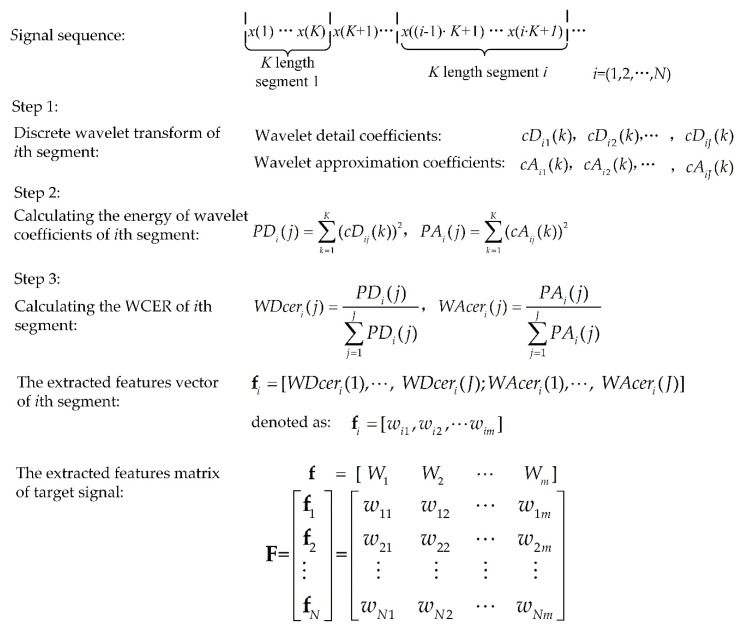
Process of feature extraction using wavelet coefficient energy ratio (WCER).

The feature vector should be extracted after a specified length of the signal sequence is acquired. Assuming the feature of the target signal sequence is extracted every *K* points, the target signal that is acquired is divided into *N* segments. The process of feature extraction based on WCER is shown in Figure 2.

Figure 2 shows the process of feature extraction based on WCER. *J* is the desired wavelet decomposition depth and *K* is the length of each segment of the signal. *cD_j_*(*k*) (*k* = 1, 2, …, *K*) represents the wavelet detail coefficient and *cA_j_*(*k*) represents the wavelet approximation coefficient of the *j*th level. **F** represents the feature matrix of the target signal. **f** = [*W*_1_, *W*_2_, …, *W_m_*] represents the feature vector and **f*_i_*** is the feature vector of the *i*th segment of the target signal. *W_m_* is the *m*th variables of the feature vector, **f**, and [*w*_1*m*_, *w*_2*m*_, …, *w_Nm_*] is the observation sequence of the variable *W_m_*.

If the sensors acquire acoustic and seismic signals of targets at a sampling rate of 4096 Hz and the signal generated by the target lasts 10 s, then the sensor node acquired 40,960 points sequence. With the *K* set to 512, then the acquired signal sequence is divided into 80 segments and every 512 points is taken as a segment for feature extracting using the WCER method. This means point 1 to point 512 is the first segment, point 513 to point 1024 is the second segment, and the entire data sequence is segmented in this way.

Subsequently, we utilize the feature extraction method, based on the WCER, to process the real-world acquired signal of vehicles in WSNs. The datasets were gathered from a real-world experiment, the third SensIT situational experiment [12]. In the experiment, 75 sensor nodes with acoustic, seismic, and infrared sensing capability were located at the Marine Corps Air Ground Combat Center. Testing runs were performed by driving different kinds of vehicles across the testing field. Four target vehicle classes were used: Assault amphibian vehicle (AAV), main battle tank, high-mobility multipurpose wheeled vehicle, and dragon wagon (DW). The acoustic and seismic data of each run were recorded and the sampling rate of the signals was 4096 Hz. There were three paths in the experimental area for the vehicles to pass and a testing run was accomplished by driving the vehicle on the path. A series of numbers was utilized to indicate these runs, e.g., DW3, DW4, DW5. The acoustic and seismic data of each run were recorded by the sensor nodes deployed at the side of the road. The details of the experiment are described in the paper mentioned above.

*K*, which is also the length of the segment of the target signal sequence, was set to 512 and the desired wavelet transform depth, *J*, was set to 8. In addition, a nearly orthogonal design of a bi-orthogonal filter bank, shown in Table 1, was utilized as the orthogonal filter bank to decompose the signal sequence.

Subsequently, we utilize the feature extraction method, based on the WCER, to extract the acoustic and seismic features of AAV3 runs.

**Figure 3:**
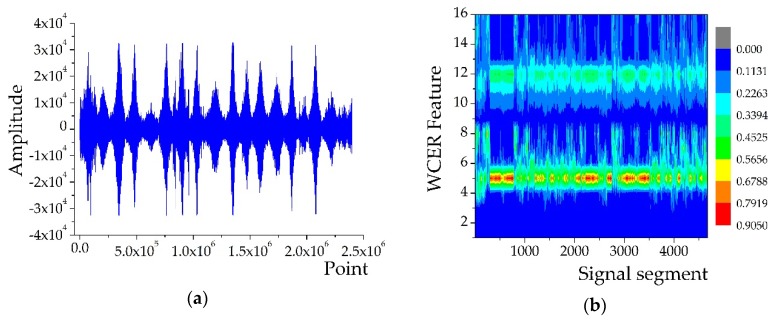

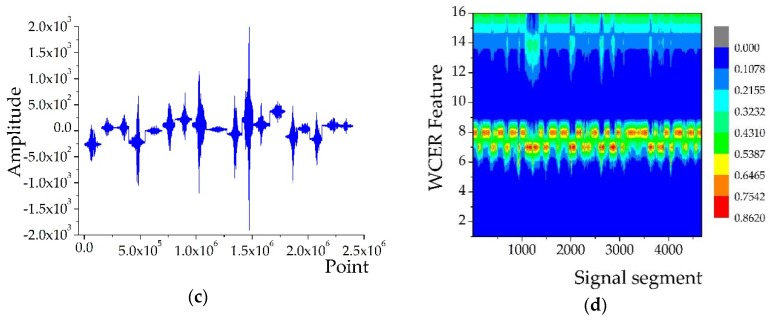
WCER features of AAV3 signals: (**a**) Acoustic signal of AAV3 acquired from 18 sensor nodes; (**b**) features extracted from the acoustic signal of AAV3; (**c**) seismic signal of AAV3 acquired from 18 sensor nodes; and (**d**) features extracted from the seismic signal of AAV3.

In the AAV3 run, the acoustic and seismic signals of AAV were sensed and acquired by 18 sensor nodes in the WSN monitoring system. All the acquired signal sequences were divided into 4676 segments at intervals of 512 points for feature extraction. Figure 3b shows the extracted features of the segmented acoustic signal sequence using the WCER method and Figure 3d shows the extracted seismic features. The horizontal axis represents the number of signal segments, which also could be considered as a set of observations of a certain feature variable, while the vertical axis represents the WCER of each segmented signal.

## 3. Feature Vector Simplification Using Hierarchical Cluster Method

The acoustic and seismic signal features of AAV3, shown in Figure 3, indicate that differences between parts of the WCER feature variables are evident and can reflect the target characteristic, whereas the differences between the remaining feature variables are insignificant. Simplifying the feature vector will remove the redundant variables in the feature vector, which can decrease the computational complexity of the subsequent target classification and effectively reduce the time consumption of the process of target classification. The hierarchical cluster method [21] and principal component analysis (PCA) method [22] are two effective approaches to reduce the dimensions of the feature matrix.

### 3.1. Hierarchical Cluster Method

The hierarchical cluster method can aggregate variables into several clusters based on their similarity. After determining representative variables from aggregated clusters to reduce the number of variables, the dimension of the feature vector will be reduced. The feature vector of the signal obtained using the WCER method is **f** = [*W*_1_, *W*_2_, …, *W_m_*], *m* = 2*J*, and *J* is the desired wavelet decomposition depth. The observation matrix of the feature vector of the target signal is denoted as **F**, whose column vector is the data sequence of the variable in the feature vector, **f**. By analyzing the sample observation matrix, the main variables in the feature vector are selected to realize its dimensionality reduction.

The hierarchical cluster method utilizes the distance between variables in the feature vector, **f,** and between clusters in the process of clustering to quantify the similarities between variables and the clusters. First, a pair of the nearest feature variables is selected to be merged into a new class according to the calculated feature variable distance matrix, **D**, expressed in Equation (7), then the two closest clusters are merged into a class. This process is repeated until a preset number of clusters is achieved:

(7) $$D=\left[\begin{array}{cccc} 0 & & & \\ dist(W_{2},W_{1}) & 0 & & \\ \vdots & \vdots & 0 & \\ dist(W_{2J},W_{1}) & \cdots & dist(W_{2J-1},W_{1}) & 0 \end{array}\right]$$

where *dist* (*W_u_*,*W_v_*) represents the distance between the variable, *W_u_*, and the variable, *W_v_*. *W_u_* is the *u*th variable in the feature vector and *W_v_* is the *v*th variable.

**Figure 4:**
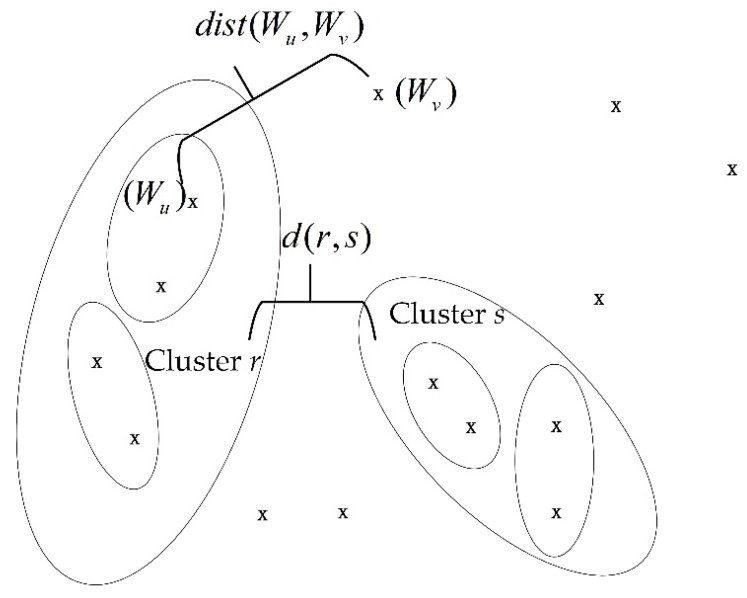
Distance between variables and between clusters.

Figure 4 shows the distance between variables in the feature vector and between clusters in the clustering process. *d (r*,*s*) represents the distance between the cluster, *r*, and the cluster, *s*.

The distance between the variables, *W_u_* and *W_v_*, is calculated using their correlation coefficient and the calculation method is expressed in Equation (8).

(8) $$dist(W_{u},W_{v})=1-R(W_{u},W_{v})=1-\frac{C(W_{u},W_{v})}{\sqrt{C(W_{u},W_{u})C(W_{v},W_{v})}}$$

where *R* (*W_u_*,*W_v_*) represents the correlation coefficient of *W_u_* and *W_v_*. C(*W_u_*,*W_v_*) is the covariance of *W_u_* and *W_v_*, and it is calculated as follows:

(9) $$C(W_{u},W_{v})=E[(W_{u}-E(W_{u}))\cdot (W_{v}-E(W_{v}))]$$

After obtaining the distance matrix, we must also calculate the distance between the clusters in the clustering process. In this study, the unweighted pair grouping method with arithmetic mean (UPGMA) [23] was utilized to calculate the distance between clusters. UPGMA is a widely used bottom-up hierarchical clustering method that defines cluster similarity [24] in terms of the average pairwise distance between all the objects in two different clusters. If the two clusters in the clustering process are the cluster, *r*, and the cluster, *s*, the formula for calculating the distance between them utilizing the UPGMA is expressed in Equation (10).

(10) $$d\left(r,s\right)=\frac{1}{n_{r}n_{s}}\overset{n_{r}}{\underset{p=1}{\sum }}\overset{n_{s}}{\underset{q=1}{\sum }}dist(W_{rp},W_{sq})$$

where *n_r_* is the number of objects in cluster *r* and *n_s_* is the number of objects in cluster *s*. *W_rp_* is the *p*th subject in cluster *r* and *W_sq_* is the *q*th subject in cluster *s*.

### 3.2. PCA Method

The PCA method is another efficient approach to reduce the dimension of the feature vector. This approach reduces the dimensions of the feature by extracting several factors that contribute the most to signal differences in the observation matrix of the target feature vector.

The feature vector of the signal is **f** = [*W*_1_, *W*_2_, …, *W_m_*] and the column vector, [*w_i_*_1_, *w_i_*_2_, …, *w_im_*], in the feature matrix, **F**, is the data sequence of the variable, *W_i_*, in the feature vector, **f**. The variable should be normalized according to Equation (11) before performing principal component analysis.

(11) $${\tilde{W}}_{i}=\frac{W_{i}-\mu_{i}}{s_{i}}$$

where *μ_i_* is the mean and *S_i_* is the standard deviation of the sequence of the variable, *W_i_*. After calculating the correlation coefficient of the variables in the feature vector, the correlation coefficient matrix can be achieved.

(12) $$C=\left[\begin{array}{ccc} R({\tilde{W}}_{1},{\tilde{W}}_{1}) & \cdots & R({\tilde{W}}_{1},{\tilde{W}}_{m})\\ \vdots & \ddots & \vdots \\ R({\tilde{W}}_{m},{\tilde{W}}_{1}) & \cdots & R({\tilde{W}}_{m},{\tilde{W}}_{m}) \end{array}\right]$$

Then, all the eigenvalues, *λ_i_* (*i* = 1, 2, …, *m*), of the correlation coefficient matrix, **C**, and the corresponding eigenvectors, [*u_i_*_1_, *u_i_*_2_, …, *u_im_*], are calculated. Then the eigenvectors consists of *m* new indicator variables shown as follows:

(13) $$\{\begin{array}{c} y_{1}=u_{11}\cdot {\tilde{W}}_{1}+u_{12}\cdot {\tilde{W}}_{2}+\cdots +u_{1m}\cdot {\tilde{W}}_{m}\\ y_{2}=u_{21}\cdot {\tilde{W}}_{1}+u_{22}\cdot {\tilde{W}}_{2}+\cdots +u_{2m}\cdot {\tilde{W}}_{m}\\ \cdots \cdots \cdots \\ y_{m}=u_{m1}\cdot {\tilde{W}}_{1}+u_{m2}\cdot {\tilde{W}}_{2}+\cdots +u_{mm}\cdot {\tilde{W}}_{m} \end{array}$$

where *y_i_* is the *i*th principal component. The contribution rate of the principal component, which means how much the new indicator variable contributes to the difference of the target features, is calculated from its corresponding eigenvector:

(14) $$\;b_{k}=\frac{\lambda_{k}}{\sum_{i=1}^{m}\lambda_{i}}\;k=1,2,\cdots ,m$$

When the sum of the former *P* contribution rates of the of new indicator variables is approximately 1 (usually 0.9 or 0.95), the former *P* indicator variables, [*y*_1_, *y*_2_, …, *y_P_*], are selected as the new features vector instead of the original *m* features.

### 3.3. Comprasion of Two Feature Simplification Methods

To show the performance of these two methods, we calculated the time consumption of the signal feature extraction using the methods for feature dimensionality reduction. We also calculated the classification accuracy of the features after reducing the dimensions using two methods. The acoustic feature data of AAV3 and AAV4 after dimension reduction were used to train the SVM classifier. Subsequently, we used the trained classifier to classify the dataset of the other 15 runs (AAV5–AAV11, DW5–DW12) after their acoustic feature data were simplified using the two methods. The classification accuracy is denoted as *Ac*. We evaluated the accuracy as follows:

(15) $$Ac=\frac{\#\mathrm{correctly}\;\mathrm{classified}\;\mathrm{data}}{\#\mathrm{total}\;\mathrm{testing}\;\mathrm{data}}\times 100\%$$

The classification accuracy of the *i*th dataset is denoted as *Ac*(*i*) (*i* = 1, 2, …, *M*) and the average classification accuracy *Aca* is:

(16) $$Aca=\frac{1}{M}\overset{M}{\underset{i=1}{\sum }}Ac\left(i\right)$$

where *M* is the number of datasets, acquired from different targets, that have been classified.

A comparison of the time needed and classification accuracy when using the hierarchical clustering and principal factor analysis methods to simplify features is shown in Table 2. **fa_HC** represents the acoustic features of the target after being simplified by hierarchical clustering and **fa_PCA** represents the acoustic features of the target after being simplified by principal factor analysis. It can be seen from Table 2 that after training the classifier, when using hierarchical clustering to reduce the dimensions of the feature, the feature extraction time is slightly less than when using principal factor analysis and the classification accuracy is higher. Therefore, the hierarchical clustering method was chosen for simplifying the feature vector.

### 3.4. Feature Vector Simplification Using Hierarchical Clustering

The acoustic and seismic signal features of AAV3 were considered as examples for simplifying the variables in the feature vectors using the hierarchical clustering method.

**Figure 5:**
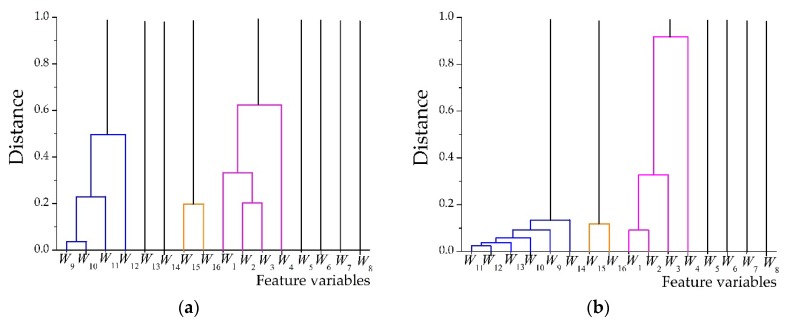
Hierarchical clustering results of feature vectors of AAV3: (**a**) Result of acoustic feature vector; (**b**) result of seismic feature vector.

Figure 5 shows the clustering results of the feature vectors of AAV3. Eight-level wavelet decomposition of the acoustic and seismic signals of AAV3 yields two feature vectors with 16 variables. The acoustic feature vector is denoted as **fa** and the seismic feature vector is denoted as **fs**. After clustering variables with a distance less than 1, the variables with large differences in the feature vectors are retained and the variables with small differences are aggregated into a cluster.

The 16 variables in the acoustic feature vector, (${\mathrm{fa}=[W}_{1},\cdots {,W}_{16}]$), were reduced to 9 after feature vector simplification. The variables, *W*_1_, *W*_2_, *W*_3_, and *W*_4_, were aggregated into a cluster and replaced with the variable, *W*_4_. The variable, *W*_12_, was utilized instead of the variables, *W*_9_, *W*_10_, *W*_11_, and *W*_12_, after they were aggregated into a cluster. Further, the variables, *W*_15_ and *W*_16_, were aggregated into a cluster and the variable, *W*_15_, was subsequently utilized instead.

The 16 variables in the acoustic feature vector, (${\mathrm{fs}=[W}_{1},\cdots {,W}_{16}])$), were reduced to 7. The variables, *W*_1_, *W*_2_, *W*_3_, and *W*_4_, were aggregated into a cluster and replaced with the variable, *W*_4_. The variable, *W*_14_, was utilized instead of the variables, *W*_9_, *W*_10_, *W*_11_, *W*_12_, *W*_13_, and *W*_14_, after they were aggregated into a cluster. Further, the variables, *W*_15_ and *W*_16_, were aggregated into a cluster and the variable, *W*_15_, was subsequently utilized instead.

The simplified acoustic and seismic feature vectors are denoted as **fa**new and **fs**new, where **fa**new = [*W*_4_, *W*_5_, *W*_6_, *W*_7_, *W*_8_, *W*_12_, *W*_13_, *W*_14_, *W*_15_], which corresponds to [*WDcer*(4), …, *WDcer*(8); *WAcer*(4), …, *WAcer*(7)]. **fs***new* = [*W*_4_, *W*_5_, *W*_6_, *W*_7_, *W*_8_, *W*_14_, *W*_15_], which corresponds to [*WDcer*(4), …, *WDcer*(8); *WAcer*(6), *WAcer*(7)].

The results of using simplified features for target classification will be discussed in Section 5.1.

## 4. Acoustic–Seismic Mixed Feature

After simplifying the features, we utilized the simplified acoustic and seismic features of the signal to classify the target.

### 4.1. Support Vector Machine Classifier

The commonly used classification methods are k-nearest neighbor (KNN) [25], decision tree (DT) [26], naive Bayes (NB) [27], and support vector machine (SVM). We used the acoustic feature data of AAV3 and AAV4 to train the classifiers and classify the acoustic feature data of the other 15 runs (AAV5–AAV11, DW5–DW12). Then, average classification accuracy and time needed were recorded to compare the performance of these methods in vehicle classification.

Table 3 shows the average classification accuracy and average time needed for different classification methods. The results indicate that KNN has the highest accuracy, but the classification takes too much time. The time needed for the DT and NB methods is very short, but their classification accuracy is lower than that of the KNN and SVM.

In summary, the SVM method has satisfactory classification accuracy and acceptable time needs. Therefore, the SVM algorithm was used as a classifier to validate the performance of the proposed WCER feature extraction method before and after feature simplification.

The SVM classification algorithm was performed by using the C support vector classifier (C-SVC) shared in LIBSVM [28], a commonly used SVM software library.

The principle of classification using SVM is to consider a multivariate feature as an independent point in a multidimensional space and, thereafter, utilize the training data to determine an optimal hyperplane to classify the independent points. Summarized mathematically, C-SVC is aimed at solving the following optimization problem:

(17) $$min(\frac{1}{2}z^{T}z+R\overset{N_{t}}{\underset{i=1}{\sum }}\varepsilon_{i})$$

under the following constraints:

(18) $$1-\varepsilon_{i}-y_{i}\left(z^{T}\phi \left(f_{i}\right)+c\right)\leq 0,\;\varepsilon_{i}\geq 0,\;i=1,\cdots ,N_{t}$$

where *N_t_* is the number of training vectors, **f***_i_* is the sample feature vector for training, and *y_i_* is the class label corresponding to the feature vector. Function, $\phi {(f}_{i})$, maps the feature vector, **f***_i_*, to a high-dimensional space and *R* is the regularization parameter, which is set to 1 in this study. Usually, we solve the following dual problem:

(19) $$min(\frac{1}{2}\alpha^{T}Q\alpha -e^{T}\alpha )$$

under the following constraints:

(20) $$y^{T}\alpha =0,\;0\leq \alpha_{i}\leq R,\;i=1,\cdots ,N_{t}$$

where *e* = [1, …, 1]*^T^* is the unit vector. **Q** is an *N_t_* by *N_t_* positive semi-definite matrix.$Q_{ij}{=y}_{i}y_{j}{K(f}_{i}f_{j})$, and $K\left(f_{i}f_{j}\right)=\phi {\left(f_{i}\right)}^{T}\phi {(f}_{j})$ is the kernel function. Moreover, the function for classifying a feature, **f**, is as follows:

(21) $$\mathrm{sgn}\left(z^{T}\phi \left(f\right)+c\right)=\mathrm{sgn}(\overset{N_{t}}{\underset{i=1}{\sum }}y_{i}\alpha_{i}K\left(f_{i}f\right)+c)$$

The label of the class is 1 or −1 for representing different kinds of targets, which indicates that $y_{i}\in \{1,-1\}$. Furthermore, we utilized a radial basis function kernel [29], with the following format:

(22) $$K\left(f_{i},f_{j}\right)=\exp(-\gamma \left\|f_{i}-f_{j}\right\|)$$

where the parameter, *γ*, in the kernel function is set to 0.5.

### 4.2. Vehicle Classification Using SVM Classifier

We extracted features from the acoustic and seismic signal data collected from 18 sensor nodes in runs AAV3, AAV4, DW3, and DW4. These extracted features were used to train the SVM classifier. Subsequently, we used the trained classifier to classify the dataset of the other 15 runs (AAV5–AAV11, DW5–DW12) before and after simplifying the acoustic and seismic WCER features.

The classification results, listed in Table 4, show that the classification accuracy of AAV is high, but that of DW was unsatisfactory, when classifying the vehicles using simplified acoustic features. By contrast, the classification accuracy of AAV was low and that of DW was satisfactory when the simplified seismic features were used. Therefore, we mixed acoustic and seismic features of the target signal to achieve satisfactory classification accuracy for both AAV and DW. The acoustic–seismic mixed features of the target signal can be described by the vectors, **f**mix and **f**mix = [**fa**new; **fs**new].

**Figure 6:**
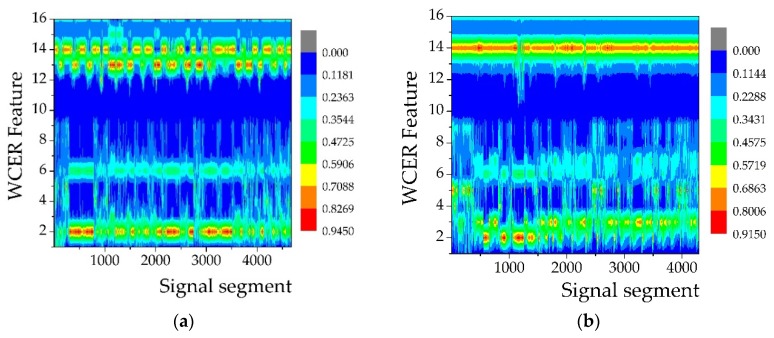
Acoustic–seismic mixed features of different vehicles: (**a**) AAV3; (**b**) DW3.

Figure 6 shows the acoustic–seismic mixed features of signals acquired from AAV3 (Figure 6a) and DW3 (Figure 6b). The results of using acoustic–seismic mixed features for target classification are discussed in Section 5.2.

## 5. Performance of WCER Method

As there is no effective approach to directly evaluate the performance of the proposed WCER feature extraction method, we evaluated the method indirectly by comparing the performance of an SVM classifier using different features.

### 5.1. Effects of Feature Simplification and Mixed Features on Classification Accuracy

To study the performance when using simplified features for target classification, we compared the classification accuracy obtained using the features before and after simplification. The features extracted from runs AAV3, AAV4, DW3, and DW4 were utilized to train the SVM classifier before and after simplification. Subsequently, the trained classifier was used to classify the target using the features extracted from the dataset of the other 15 runs (AAV5–AAV11, DW5–DW12) before and after simplification. The set of parameters of the classifier is described in detail in Section 4. The classification accuracy obtained by using the acoustic–seismic mixed features was calculated similarly for studying the effect of feature mixing on vehicle classification.

Table 5 shows the classification accuracy obtained by using different feature vectors based on the WCER method. **fa** (**fs**) indicates that the targets were classified using the feature extracted from the acoustic (seismic) signal of the target without simplification and **fa**new (**fs**new) indicates that the targets were classified using the feature extracted from the acoustic (seismic) signal of the target after feature simplification. **f**mix represents the classification of vehicles using acoustic–seismic mixed features of the target signal.

To further clarify the classification accuracy obtained by using different extracted features, Table 4 summarizes the average classification accuracy.

We also calculated the time consumption of target classification using these features to compare the efficiency of using different features. Two types of time use were calculated to compare the efficiency: Time consumption of the feature extraction procedure and the time needed for subsequent target classification using the extracted features. To compare the time consumption of the feature extraction step, we utilized the feature extraction methods to process the dataset of 15 runs (AAV5–AAV11, DW5–DW12) and calculated the average time consumed by feature extraction in each run. Subsequently, the average time consumption of target classification using these feature vectors was recorded to study the effect of using different feature vectors on the time consumption of the target classification.

The average classification accuracy and average time consumption when using different feature vectors are shown in Table 6. The results show that the WCER feature extraction method can effectively extract the target features from the target signals. When using the target acoustic and seismic features extracted using the WCER method to classify vehicles, the classification accuracy reached approximately 67%. Feature simplification can reduce the time consumption of feature extraction and classification, while having no effect on target classification accuracy. Compared with the use of feature vectors before simplification, the time consumption after feature simplification decreased slightly and the time consumption of target classification decreased by almost half. The average classification accuracy obtained by using simplified WCER features of the acoustic signal was 0.56% lower than that obtained by using unsimplified features, and the classification accuracy decreased by 0.38% after the features extracted from the seismic signal were simplified. This is mainly because the removed parts of the feature vectors in the simplification process were redundant and had no effect on the overall characteristics of the target signal.

Using mixed features can effectively improve the target classification accuracy. Compared with the use of acoustic or seismic features, classification accuracy was improved by approximately 12% when the acoustic–seismic mixed feature of the target signal was used.

### 5.2. Comparison with FFT-Based Feature Extraction Method

The experimental results in Section 5.1 show that the WCER feature extraction method can effectively extract the characteristics of the target signal and that the feature mixing approach can improve classification accuracy. However, whether the performance of this feature extraction method is better than that of the existing methods is yet to be determined.

The FFT-based feature extraction method mentioned in Section 1 is a mature and commonly used feature extraction method. To extract the features of the target signal, the FFT of the acoustic signals is computed every 512 points. Subsequently, the averages of every two points of the first 100 FFT values that have been normalized are used as the extracted features of 50 dimensions for target classification. We used this feature extraction method to compare with the proposed hybrid feature extraction method.

The data of runs AAV3, AAV4, DW3, and DW4 were used for training and the features extracted from the dataset of the other 15 runs (AAV5–AAV11, DW5–DW12) were classified by the trained SVM classifier.

The average time consumptions of the FFT-based and the WCER feature extraction methods were calculated to compare the efficiency of these methods.

As shown in Table 7 and Table 8, the average classification accuracy of the WCER method was 2.88% higher than that of the FFT-based method when the features extracted from the target acoustic signal were used. This is mainly because only the energy of the low-frequency band of the target signal is extracted by the FFT-based method and utilized for target classification. Compared with the FFT-based method, the proposed WCER feature extraction method utilizes all the signal spectrum energy characteristics to classify targets. We also observed that the time consumed by the proposed WCER method to extract features from the target acoustic signal is less than that of the FFT-based method because wavelet decomposition of the signal sequence takes less time than the FFT operation of the same signal sequence. Moreover, the number of variables in the signal feature extracted by the WCER method is less than the number of feature variables extracted by the FFT-based method, resulting in less time spent on subsequent target classification when using the proposed WCER method.

Compared with the FFT-based feature extraction method, average classification accuracy is improved by 15.5% when using acoustic–seismic mixed features. At the same time, the time consumption of target feature extraction is increased significantly. This is because the increased number of calculations leads to more time spent as the WCER method requires extraction of not only the features of the target acoustic signal, but also of the seismic features of the same target when using the acoustic–seismic mixed features.

## 6. Conclusions

**Figure 7:**
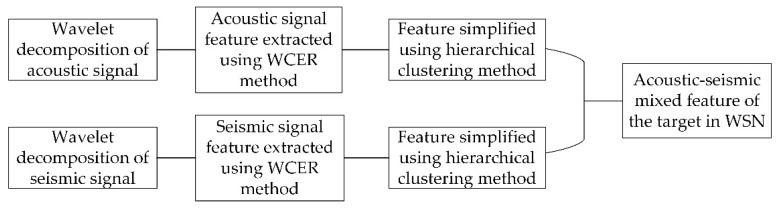
Flowchart of the acoustic–seismic mixed feature extraction method based on WCER.

In this paper, we proposed a novel feature extraction method named WCER based on WT for vehicle classification in WSNs. The acoustic-seismic mixed feature extraction method is presented after the acoustic and seismic WCER features of the target signal were simplified using the hierarchical clustering method. The flow of the proposed method for extracting acoustic–seismic mixed features of the target is shown in Figure 7. Finally, we studied the target classification accuracy and time consumption of the proposed feature extraction method. The experiment results show that classification accuracy of the targets detected in WSN is effectively improved by using the acoustic–seismic mixed feature of the target.

Another advantage of using the wavelet-based feature extraction method is that the wavelet coefficients can be processed using the thresholding method [30] for denoising before calculating the WCER of the target signal. Thus, the combination of the denoising and feature extraction methods can effectively reduce the time consumption of the entire signal processing of WSN from denoising to target classification.

## Figures and Tables

**Table 1:** Coefficients of quasi-orthogonal bi-orthogonal filters.

*l*	h_0_(*l*)	g_0_(*l*)	*l*	h_0_(*l*)	g_0_(*l*)
			0	0.5613	0.5601
−1	0.2865	−0.2961	1	0.3030	−0.2961
−2	−0.0432	−0.0470	2	−0.0508	−0.0470
−3	−0.0465	0.0552	3	−0.0582	0.0552
−4	0.0166	0.0220	4	0.0244	0.0220
−5	0.0055	−0.0105	5	0.0112	−0.0105
−6	−0.0027	−0.0058	6	−0.0064	−0.0057
−7	0	0.0018	7	−0.0018	0.0018
−8	0	0.0007	8	0.0008	0.0007
−9	0	−0.0003	9	0.0004	−0.0003
−10	0	0	10	0.0001	0
−11	0	0	11	0	0

**Table 2:** Average classification accuracy and time consumption of feature extraction using different simplification methods. **fa_HC**, acoustic features after hierarchical clustering; **fa_PCA**, acoustic features after principal factor analysis.

	Using fa_HC	Using fa_PCA
Average classification accuracy	66.98%	63.52%
Time consumption of feature extraction	4.1543 s	4.1809 s

**Table 3:** Average classification accuracy and time needed for different classification methods using acoustic features without simplification. KNN, k-nearest neighbor; DT, decision tree; NB, naïve Bayes; SVM, support vector machine.

	KNN	DT	NB	SVM
Average classification accuracy	71.55%	65.77%	64.29%	67.54%
Average time needed for classification	6.6225 s	0.0029 s	0.0051 s	0.4282 s

**Table 4:** Classification accuracy with different feature vectors.

**Runname**	**Usingfa**new	**Usingfs**new	**Runname**	**Usingfa**new	**Usingfs**new
AAV5	91.3609%	43.9645%	DW5	**85.4323%**	80.968%
AAV6	90.6977%	51.292%	DW6	84.5255%	88.4185%
AAV7	90.0334%	48.8294%	DW7	25.4846%	86.7161%
AAV8	91.7002%	49.4762%	DW8	29.8495%	69.5652%
AAV9	99.9269%	51.462%	DW9	6.6007%	72.7723%
AAV10	89.5207%	55.9371%	DW10	35.2547%	91.1081%
AAV11	88.8577%	42.8371%	DW11	51.8025%	94.4691%
			DW12	43.695%	85.9726%

**Table 5:** Comparison of classification accuracy using different feature vectors.

**Runname**	**Using fa**	**Usingfa**new	**Using fs**	**Usingfs**new	**Usingf**mix
AAV5	91.6568%	91.3609%	44.9704%	43.9645%	90.5917%
AAV6	91.7313%	90.6977%	51.2489%	51.292%	89.4488%
AAV7	90.6355%	90.0334%	50.903%	48.8294%	89.4314%
AAV8	92.3449%	91.7002%	51.0073%	49.4762%	90.2498%
AAV9	99.8538%	99.9269%	53.655%	51.462%	97.2953%
AAV10	90.3076%	89.5207%	56.0086%	55.9371%	84.2275%
AAV11	89.5599%	88.8577%	43.4457%	42.8371%	86.0955%
DW5	85.1034%	85.4323%	80.9211%	80.968%	79.3703%
DW6	85.1582%	84.5255%	89.2457%	88.4185%	87.6399%
DW7	26.1688%	25.4846%	85.8609%	86.7161%	71.2657%
DW8	31.1037%	29.8495%	68.7291%	69.5652%	60.9532%
DW9	7.75578%	6.6007%	72.5248%	72.7723%	49.9175%
DW10	36.6399%	35.2547%	90.4379%	91.1081%	69.3923%
DW11	52.0988%	51.8025%	94.6173%	94.4691%	72.2469%
DW12	42.913%	43.695%	85.9726%	85.9726%	75.8065%

**Table 6:** Average classification accuracy and time consumption using different feature vectors.

	**Using fa**	**Usingfa**new	**Using fs**	**Usingfs**new	**Usingf**mix
Average classification accuracy	67.54%	66.98%	67.97%	67.59%	79.60%
Average time consumption of feature extraction)	4.1696 s	4.1543 s	7.3511 s	7.2377 s	15.3392 s
Average time consumption of classification	0.4282 s	0.2761 s	0.6403 s	0.3734 s	0.3869 s

**Table 7:** Comparison of classification accuracy of different feature extraction methods.

**Runname**	**Usingf**mix	**Using FFT-Based Features**	**Runname**	**Usingf**mix	**Using FFT-Based Features**
AAV5	90.5917%	93.8462%	DW5	79.3703%	90.7895%
AAV6	89.4488%	97.7606%	DW6	87.6399%	94.7445%
AAV7	89.4314%	52.5753%	DW7	71.2657%	25.0855%
AAV8	90.2498%	74.0532%	DW8	60.9532%	40.1338%
AAV9	97.2953%	62.7924%	DW9	49.9175%	21.6172%
AAV10	84.2275%	88.7339%	DW10	69.3923%	42.8061%
AAV11	86.0955%	90.2154%	DW11	72.2469%	46.1728%
			DW12	75.8065%	40.176%

**Table 8:** Average classification accuracy and time needed for different feature extraction methods.

	**Usingf**mix	**Usingfa**new	**Using FFT-Based Features**
Average classification accuracy	79.60%	66.98%	64.10%
Efficiency (time consumption of feature extraction)	15.3392 s	4.1543 s	5.5515 s
Time consumption of classification	0.3869 s	0.2761 s	0.3749 s

## Abbreviations

The following abbreviations are used in this manuscript

WCER	Wavelet coefficient energy ratio
WSNs	Wireless sensor networks
WT	Wavelet transform
DWT	Discrete wavelet transform
FFT	Fast Fourier transform
AAV	Assault amphibian vehicle
DW	Dragon wagon
UPGMA	Unweighted pair grouping method with arithmetic mean
PCA	Principal component analysis
KNN	K-nearest neighbor
DT	Decision tree
NB	Naive Bayes
SVM	Support vector machine
C-SVC	C support vector classifier
